# Providing a conceptual framework for HIV prevention cascades and assessing feasibility of empirical measurement with data from east Zimbabwe: a case study

**DOI:** 10.1016/S2352-3018(16)30039-X

**Published:** 2016-06-27

**Authors:** Geoffrey P Garnett, Timothy B Hallett, Albert Takaruza, James Hargreaves, Rebecca Rhead, Mitchel Warren, Constance Nyamukapa, Simon Gregson

**Affiliations:** a Bill & Melinda Gates Foundation, Seattle, WA, USA; b Department of Infectious Disease Epidemiology, Imperial College London School of Public Health, London, UK; c Biomedical Research and Training Institute, Harare, Zimbabwe; d London School of Hygiene & Tropical Medicine, London, UK; e AVAC, New York, NY, USA

## Abstract

**Background:**

The HIV treatment cascade illustrates the steps required for successful treatment and is a powerful advocacy and monitoring tool. Similar cascades for people susceptible to infection could improve HIV prevention programming. We aim to show the feasibility of using cascade models to monitor prevention programmes.

**Methods:**

Conceptual prevention cascades are described taking intervention-centric and client-centric perspectives to look at supply, demand, and efficacy of interventions. Data from two rounds of a population-based study in east Zimbabwe are used to derive the values of steps for cascades for voluntary medical male circumcision (VMMC) and for partner reduction or condom use driven by HIV testing and counselling (HTC).

**Findings:**

In 2009 to 2011 the availability of circumcision services was negligible, but by 2012 to 2013 about a third of the population had access. However, where it was available only 12% of eligible men sought to be circumcised leading to an increase in circumcision prevalence from 3·1% to 6·9%. Of uninfected men, 85·3% did not perceive themselves to be at risk of acquiring HIV. The proportions of men and women tested for HIV increased from 27·5% to 56·6% and from 61·1% to 79·6%, respectively, with 30·4% of men tested self-reporting reduced sexual partner numbers and 12·8% reporting increased condom use.

**Interpretation:**

Prevention cascades can be populated to inform HIV prevention programmes. In eastern Zimbabwe programmes need to provide greater access to circumcision services and the design and implementation of associated demand creation activities. Whereas, HTC services need to consider how to increase reductions in partner numbers or increased condom use or should not be considered as contributing to prevention services for the HIV-negative adults.

**Funding:**

Wellcome Trust and Bill & Melinda Gates Foundation.

## Introduction

To halt the spread of HIV requires that efficacious treatment and preventive products or behaviours are used by those at risk. These products or behaviours are the direct mechanisms through which HIV prevention programmes exert their effect. Analyses of whether programmes are effectively preventing HIV transmission from mothers to children, and whether programmes are effectively treating patients with HIV, have used the concept of a cascade to explore the steps required to prevent infection or death.^1^, ^2^, ^3^, ^4^ This approach has powerfully illustrated what needs to be done to suppress HIV viral loads in many populations and is increasingly being used to compare the performance of prevention of mother to child transmission (PMTCT) and treatment programmes.^5^, ^6^ An HIV prevention cascade could be a similarly powerful approach to complement the treatment cascade, by defining the different steps in successful implementation of prevention interventions, providing estimates of the proportions of populations lost at each step in implementation, demonstrating points at which inefficiencies occur, and providing a framework for planning further actions.

The idea of using models to describe the steps in controlling infectious diseases was initially developed to explore the effectiveness of tuberculosis control in the 1960s.^7^ Illustrating the coverage of diagnosis and treatment was extended to HIV, and has become common, although different approaches have been applied.^6^ The characteristics of the HIV treatment cascade depend upon whether data comes from health facilities tracking patients or from population based studies where all those infected with HIV can be included.^6^ The cascade also differs according to whether it provides a cross-sectional view of the population at a particular point in time or follows cohorts longitudinally describing their progression over time through the cascade.^8^ The description of care^9^ and other HIV services,^10^ in addition to treatment in cascades, can change the steps included and has culminated in the HIV prevention, treatment, and care cascade in the WHO Consolidated Strategic Information Guidelines for HIV in the Health Sector^11^ and the HIV Cascade Framework for Key Populations.^12^ These cascades attempt to include HIV prevention, but emphasise the steps of diagnosis, linkage to treatment, treatment initiation, and viral suppression. Often HIV testing is seen as an essential gateway to both HIV treatment and prevention, and is pivotal in the HIV prevention, treatment, and care cascade.^11^ The ambition of capturing all HIV prevention interventions in one cascade is challenging because various approaches exist that focus on either HIV infected or HIV uninfected people and use alternative products and approaches such as treatment as prevention, condoms, pre-exposure prophylaxis, voluntary medical male circumcision (VMMC), and reduction in sexual partner numbers.^11^ To explore the steps required for a particular approach to prevent HIV, each intervention can be separated out and described in terms of how it can succeed or fail in the epidemiological context in which it is used. This does not imply that each intervention should be used in isolation or that each provider should focus on a single intervention, rather it describes the steps that need attention for an intervention to be a worthwhile part of HIV prevention.

**Research in context**

**Evidence before this study**

The HIV or cascade treatment cascade has become a tool to evaluate programme performance describing the steps required for those HIV infected to be successfully virally suppressed. In addition to its advocacy role the HIV treatment and care cascade can act as a guide to improving programme performance. The HIV treatment, prevention, and care cascade has started to be discussed as a comprehensive HIV cascade. However, to date such a cascade has yet to be fully populated with data. To explore the use of cascades in the HIV field PubMed was searched using the terms “HIV treatment cascade”, “HIV prevention cascade”, “HIV care cascade”, with last search on Feb 23, 2016.

**Added value of this study**

This study describes how HIV prevention cascades might be considered in theory and then proceeds to use field data from rural Zimbabwe to generate HIV prevention cascades for voluntary male medical circumcision and for condom use and partner reduction after testing and counselling. The study illustrates the use of prevention cascades and provides a template for prospective studies to develop the practical use of the concept further in planning, implementing, and evaluating HIV prevention interventions.

**Implications of the available evidence**

In this population in rural Zimbabwe HIV prevention interventions have improved but are having little impact. VMMC was limited by supply in 2009–11, but is increasingly limited by demand. HIV testing and counselling has little effect because men and particularly women do not change their behaviour. Perception of risk is a barrier to uptake on interventions particularly among men.

For cascades to be useful, in addition to a theoretically justified description of HIV treatment and prevention, practical sources of data need to be available with which to populate the cascades. In this paper, we propose a new conceptual framework for HIV prevention cascades and illustrate its application with data collected in a large-scale general-population cohort in eastern Zimbabwe.

## Methods

### Conceptual framework

**Figure 1:**
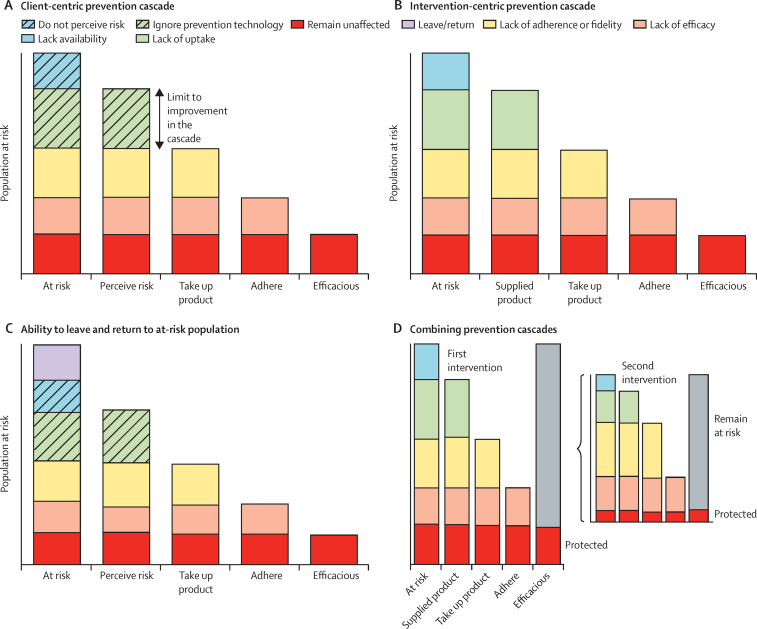
Generic conceptual HIV prevention cascades applied to the population who would otherwise acquire HIV or are at risk of acquiring HIV (A) Steps to prevention taking the clients' perspective and their perception of risk into account. (B) Steps to prevention taking the intervention perspective and whether there is supply of the product available. (C) Including the possibility that those at risk can move into a not at risk population and vice versa. (D) Combining two prevention cascades where the population at risk for the start of the second intervention is the population that remain at risk after the application of the first intervention.

To be effective, prevention products or protective behaviours have to be used by those who can benefit from them, and that use depends upon delivery, acceptance, and adoption by the appropriate population as well as adherence. The treatment cascade looks at the HIV infected population and the steps as they progress to viral suppression. An analogous client-centric prevention cascade could explore how those at risk of acquiring HIV (as a starting point) could avoid infection by perceiving that risk and acting on that risk by both adopting (ie, taking up) and adhering to an efficacious prevention approach (figure 1). An alternative would be an intervention-centric approach, in which programme staff identify a priority population, make an intervention available, see the uptake of that product, and see that it is used appropriately and that it is efficacious (figure 1). These prevention cascades progress from a population at risk of acquiring infection over a given period of time. The steps show the reduction in effect, and, as a corollary, the effect that improving each step can achieve. Efficacy, defined as the according-to-protocol estimate of effect of an intervention in an individual randomised controlled trial, is the last step in the cascade and is of limited importance if an intervention is not delivered, adopted, and adhered to. An intervention needs to be made available through supply of products, information, or procedures to the population at risk. Then the priority population has to take up the products, information, or procedures. This requirement for supply and then demand could be illustrated the other way round with demand being necessary and then limited by supply. Supply and demand are inextricably linked, with both required for effective prevention programmes.

To describe the prevention cascades defining the population at risk of acquiring infection is an important first step. Theoretically, if we could accurately identify those that would acquire the infection without the intervention, then those lost at each step would acquire infection and the population left after all the steps of the cascade would be those staying uninfected. However, we can only identify those with a risk of infection at the start, so losses would be those remaining at risk and the end would represent those no longer at risk. Better focus on priority populations would imply a smaller population that could benefit from prevention to be included in programmes and a smaller number in the prevention cascade. The same proportional reductions in effect size through lack of supply, take-up, adherence, or efficacy apply to those who have a risk of acquiring infection or those who would actually acquire infection over a period. Thus, the starting for the prevention cascade could be the predicted incidence of infection without the intervention, and the probable number of infections occurring with the intervention would be determined by the number dropping out along the cascade. Risk behaviour is not constant so one could see the denominator at risk population changing over time. Acknowledging the not-at-risk population and allowing movement from not at risk to at risk may be possible (figure 1). This implies that individuals are removed entirely from potential risk of exposure; alternatively, their risk could have been reduced through successful use of a different prevention approach, which would mean that the population at risk of acquiring HIV at the start of one prevention cascade is a function of other prevention cascades (figure 1).

### Data sources

To explore the potential of cascades to track the implementation and impact of HIV prevention interventions, we used data from a general population open cohort HIV serosurvey done in Manicaland, eastern Zimbabwe, the Manicaland HIV/STD Prevention Project. Details of the cohort have been published previously.^1^, ^2^, ^13^, ^14^ In brief, a baseline survey and five rounds of follow-up have been done in a random sample of between 8000 and 15 000 adults recruited in a prospective census in 12 sites (eight sites in round six) in small towns, agricultural estates, roadside settlements, and rural villages spread across three districts in Zimbabwe's Manicaland province. Numbers varied between rounds because of demographic changes in the underlying population and changes in the sampling fraction following fluctuations in funding. The data collected in each round included information on HIV infection status, sexual behaviour (collected with a method to reduce social desirability bias),^15^ and availability, uptake, and adherence to HIV services such as VMMC, HIV testing and counselling (HTC), and so on. HIV prevalence in the study areas declined from 24·2% in 1998–2000 to 14·5% in 2012–13; HIV incidence was 0·80% in 2006–11 and 0·67% in 2009–13.

Here, we used data from the eight sites covered in both of the two most recent rounds of follow-up of the Manicaland general population cohort study (2009–11 and 2012–13). Eligibility was restricted to adults aged 15–54 years who were resident in and had stayed in a household in the study areas for at least four nights in the last month. Descriptive statistics were calculated for uptake of each HIV prevention approach by the study population at each round. Intervention-centric HIV prevention cascades (based on local availability of services) and client-centric HIV prevention cascades (based on respondents' perceived personal risk of acquiring HIV infection) were constructed for all men and women, separately, who reported ever having had sex. Further intervention and client centred HIV prevention cascades were constructed for those reporting casual sexual partners in the 3 years before each survey to investigate the effect of HTC in reducing risk through sexual partner reduction and increased condom use. Our aim was to see how much impact HTC was having on risk behaviour and HIV incidence and test whether in its current form in this community it should be classed as an HIV prevention intervention.

Availability, risk perception, uptake, and adherence for each HIV prevention approach within the study population were measured with existing questions rather than questions specifically designed to explore prevention cascades. Respondents were taken to have a service available locally when they knew that the service was being provided at a facility within 20 km of their homestead. Uptake of VMMC was measured with a variable derived from the question “Have you ever been circumcised yourself?” (coded “Yes–full”, “Yes–partial”, and “No”) and a question on the person performing the circumcision (doctor, nurse, traditional healer, tribal elder). Men with partial circumcisions or circumcisions done by traditional practitioners or tribal elders were treated as uncircumcised in the HIV prevention cascades for VMMC. Uptake of HTC was measured using the question “On how many different occasions have you had an HIV test in your lifetime?” and a question on collection of most recent test results. Variables for VMMC and HTC uptake in the last 3 years were also created with responses to questions on age at circumcision and number of HIV tests taken in the previous 3 years. For HTC, variables on adherence to sexual partner reduction and increased condom use were constructed from responses to the questions “After the HIV test, did you start having more or fewer sexual partners? / use condoms more or less than before?” We have estimates of the efficacy of VMMC of 60%,^16^ but for partner reduction and condom use we have only reports about whether or not behaviour changed so have to assume by how much. For illustrative purposes, we assume a 50% reduction in risk associated with reducing partner numbers, which is commensurate with the change in risk observed in earlier studies of risk reduction in this population^13^, ^17^ and 80% reduction associated with condom use reported in systematic reviews.^18^ For the client-centric HIV prevention cascades, perceived risk among individuals currently not infected was measured using the question “If you are not infected (currently), do you think you are in danger of getting infected now or in the future?”

### Data analysis

To construct intervention-centric cascades, data were available on the number of people reporting that a service was available in their area, the number who had taken up the service. The efficacy of the intervention was estimated as described above. For circumcision adherence is not relevant, but for HIV testing and counselling adherence is represented by the proportion reporting reduced partner numbers or increased condom use. In the client-centric cascade the first reduction was based on the number reporting that they did not perceive that they were at risk of HIV and then the next step was based on whether they had taken up the intervention. To normalise the cascades and make them comparable the cascades were created for 1000 individuals with proportionate reductions at each step based on the numbers in the corresponding round of the Manicaland GPCS cohort. So for each 1000 population the number protected was first reduced by not having the service available or not perceiving risk, then reduced by the number not taking up the service, then by the number not adhering (zero in the case of circumcision), and then by the number where there would not be efficacy leaving the number who would be protected from those thousand individuals. To explore in more detail perceptions of risk we calculated age adjusted odds ratios comparing perception in risk between men and women and uptake in HTC as a function of perception of risk.

For both types of cascade, estimates of potential HIV incidence in the absence of a given intervention *I*_p_ were calculated with the formula *I*_p_ = [N / (N – *e* × N_c_)] × *I*_o_, where *I*_o_ and N are the observed (actual) HIV incidence and the sample size for the specific populations of 15–49 year olds in the relevant round of the Manicaland GPCS, N_c_ is the number of individuals in the population who take up and adhere to the service, and *e* is the efficacy of the service, which can be measured in the according-to-protocol analysis in randomised controlled trials. This estimate is based on the reduction in incidence from what would have been the case without an intervention being the product of effective coverage and efficacy for that intervention. For example if observed incidence in a population is 1% and 400 of 1000 people have adhered to an intervention with 50% efficacy then the incidence without the intervention would have been 1·25%. Details of the HIV incidence estimates observed in the Manicaland GPCS are given in the appendix. Confidence intervals were calculated for binomial outcomes using Stata SE12.

### Role of the funding source

GPG is an employee of the Bill & Melinda Gates Foundation, other than this the funder of the study had no role in study design, data collection, data analysis, data interpretation, or writing of the report. The corresponding author had full access to all the data in the study and had final responsibility for the decision to submit for publication.

## Results

In the study sites, 3·1% (95% CI 2·5–3·7) of the 3131 men interviewed and not infected with HIV in 2009–11 were circumcised: 1·8% (1·4–2·3) had received VMMC and 1·3% (0·9–1·7) had received traditional circumcision (table). VMMC coverage was somewhat higher (2·7%; 2·0–3·6) among the 1873 men who reported having passed their sexual debut. 3 years later, 6·9% (5·9–8·1) of the 2245 men interviewed in 2012–13 had been circumcised: 4·1% (3·4–5·1) for VMMC and 2·8% (2·2–3·6) for traditional circumcision.

**Figure 2:**
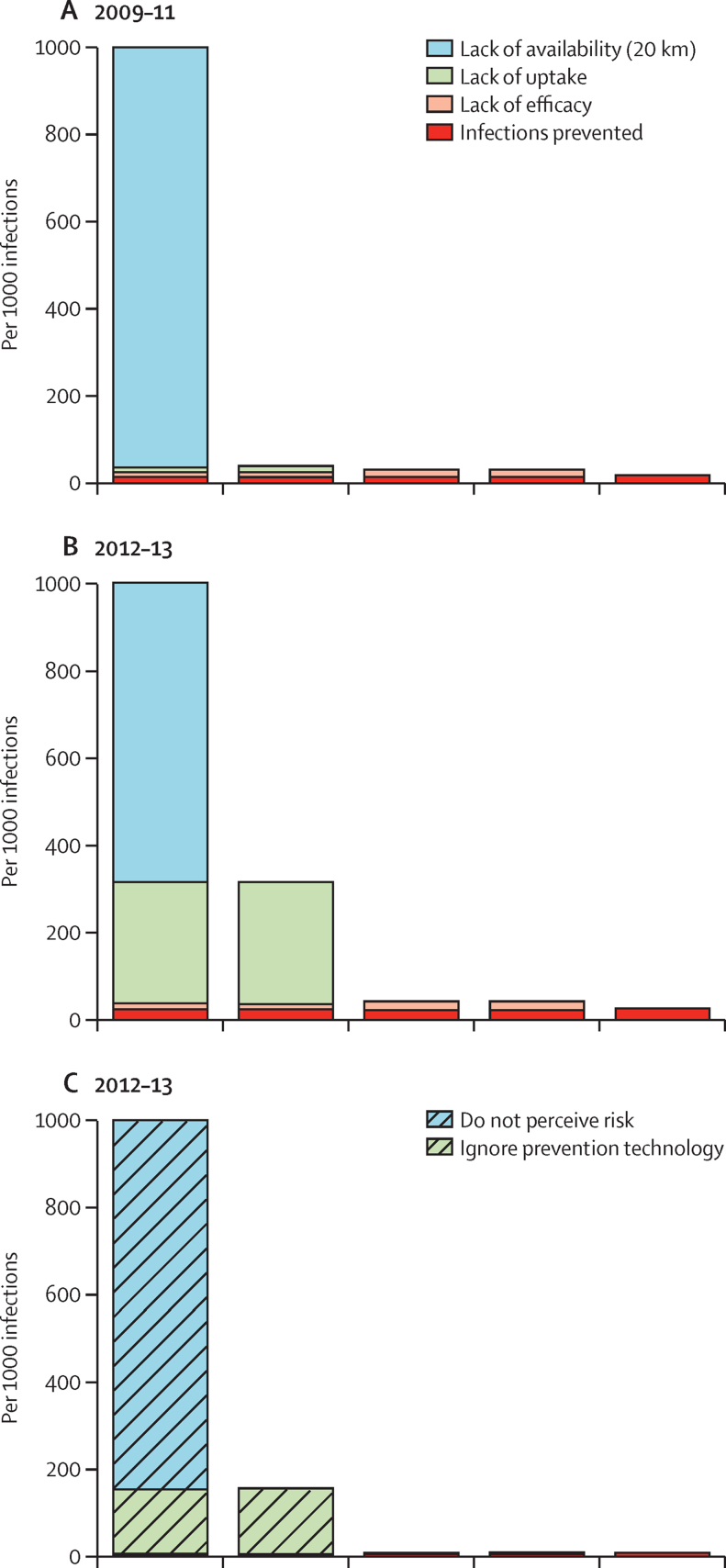
HIV prevention cascades for voluntary medical male circumcision in sexually experienced uninfected men aged 15–54 years in Manicaland, Zimbabwe (A) Cascade based on service availability for 1888 individuals in 2009–11. (B) Cascade based on service availability for 1476 individuals in 2012–13. (C) Cascade based on participant's perception of personal risk of acquiring HIV infection for 1476 individuals in 2012–13.

HIV infections occurring because of unavailability of local VMMC services were reduced from 96·4% to 68·4% over a period of about 3 years between surveys (figure 2). However, the number of infections prevented increased only modestly from 16·3 per 1000 to 22·8 per 1000 (assuming an efficacy of 60%) because of limited uptake (12·0%) among men who knew that VMMC services were locally available. In the absence of the VMMC programme, HIV incidence would have been 0·69% per person-year of exposure compared to 0·67% rate observed between 2009 and 2013 in the GPCS, a reduction of just 0·02%.

**Figure 3:**
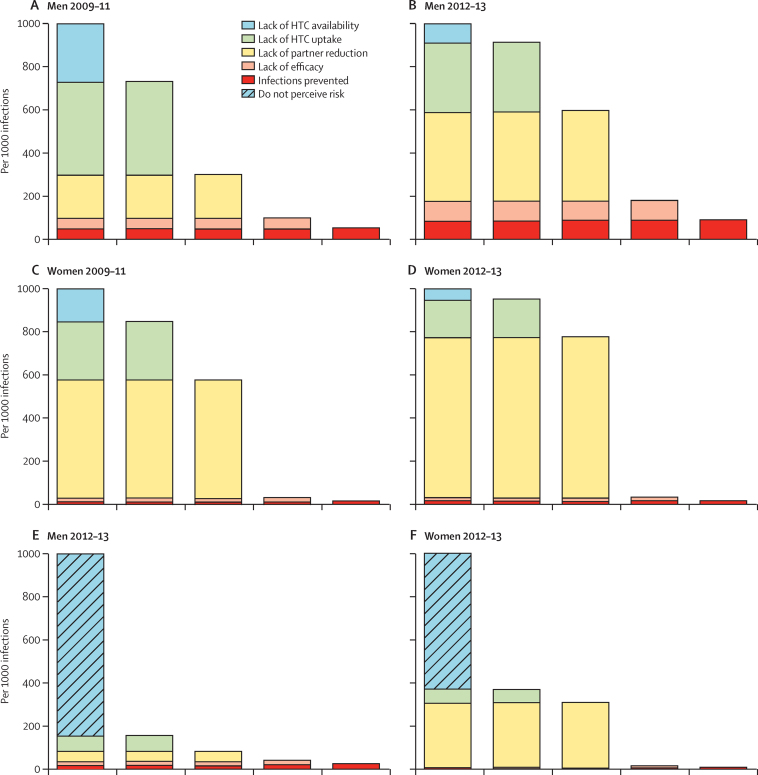
HIV prevention cascades for HIV testing and counselling and sexual partner reduction in sexually experienced uninfected adults aged 15–54 years in Manicaland, Zimbabwe (A) Cascade for 1888 men based on service availability in 2009–11. (B) Cascade for 1468 men based on service availability in 2012–13. (C) Cascade for 3793 women based on service availability in 2009–11. (D) Cascade for 2743 women based on service availability in 2012–13. (E) Cascade for 1468 men based on participant's perception of personal risk of acquiring HIV infection in 2012–13. (F) Cascade for 2743 women based on participant's perception of personal risk of acquiring HIV infection in 2012–13.

The client-centric HIV prevention cascade for 2012–13 (figure 2) shows that 85·3% (1876 of 2199) of uninfected men in Manicaland did not perceive a personal risk of HIV infection. However, even in those who did believe themselves to be at risk, uptake of VMMC services was just 4·0% (13 of 323). In 2009–11, 27% of men (867 of 3154) and 57% of women (1284 of 2270) reported ever having had an HIV test, these proportions increased to 61% (2872 of 4703) and 80% (2625 of 3297) in 2012–13 (table). In 2009–11, lack of known availability and, particularly, lack of uptake when services were known to be available, were the principal obstacles to HTC having a major effect in preventing HIV infections (figure 3). Both of these obstacles were reduced substantially by 2012–13, at which point, the main obstacle was lack of partner reduction among men who took up HTC (table). In 2009–11, HTC, by bringing about reductions in numbers of sexual partners, prevented 49 per 1000 infections and reduced HIV incidence from 0·99% (in the absence of the programme) to 0·94% per person-year. In 2012–13, the HIV prevention effect of the HTC programme almost doubled to 90 per 1000 infections, and HIV incidence was reduced from 0·74% to 0·67%.

Much of the failure to prevent HIV infections resulting from lack of partner reduction stems from the presence of men who report abstinence or monogamy before taking up HTC in the population at risk. Restricting the analysis for 2009–11 to men with one or more casual sexual partners in the previous 3 years, reported availability and uptake of HTC were unchanged, but fewer men reported lack of partner reduction (145 per 1000 compared with 200 per 1000; appendix p 7). Given their higher rate of new infections, this yielded a somewhat greater absolute reduction in HIV incidence in this group from 1·58% to 1·45%.

For women, availability of HTC services and uptake when services were known were higher than for men, but availability and particularly uptake were still problematic in 2009–11 (figure 3). Lack of uptake remained a limiting factor in 2012–13 (figure 3) but the main obstacle in HTC contributing to reductions in HIV risk for women in both rounds was lack of partner reduction (table). Even more so than for men, this reflects large numbers of sexually experienced women who reported abstinence or monogamy prior to taking up HTC and conceal the greater prevention effect in women with casual partners (appendix p 8). However, overall, the effect of HTC in reducing numbers of sexual partners only reduced HIV incidence from 0·80% to 0·79% in the general population in the 3 year period to 2012–13.

**Figure 4:**
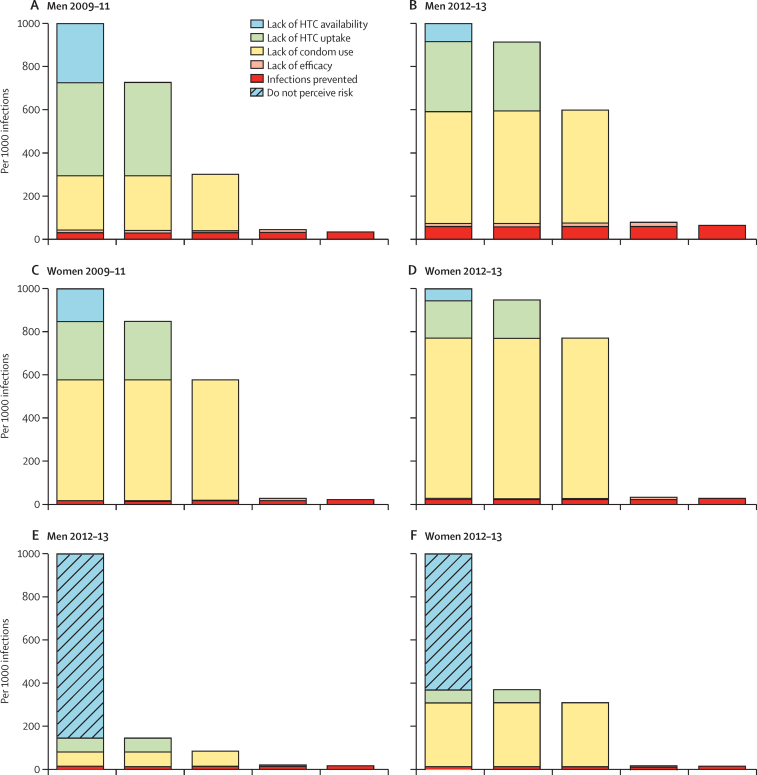
HIV prevention cascades for HIV testing and counselling and increased condom use in sexually experienced uninfected adults aged 15–54 years in Manicaland, Zimbabwe (A) Cascade for 1888 men based on service availability in 2009–11. (B) Cascade for 1468 men based on service availability in 2012–13. (C) Cascade for 3793 women based on service availability in 2009–11. (D) Cascade for 2743 women based on service availability in 2012–13. (E) Cascade for 1468 men based on participant's perception of personal risk of acquiring HIV infection in 2012–13. (F) Cascade for 2743 women based on participant's perception of personal risk of acquiring HIV infection in 2012–13.

The contributions of lack of availability and lack of uptake of HTC for condom use (figure 4) are the same as for reducing numbers of sexual partners. Fewer men report increasing condom use after HTC than report reduced sexual partners (appendix p 4; table 1), which offsets the smaller (assumed) lack of efficacy of condom use (20% vs 50%; figure 4). For example, in 2012–13, the effect of HTC in reducing HIV risk in sexually experienced men by increasing condom use was 61 per 1000 (appendix p 13) compared to 90 per 1000 (appendix p 9) achieved by reducing sexual partners.

For women, lack of condom use after HTC, once again, was the main factor contributing to the very small effect of HTC in preventing HIV infections in those otherwise at risk (figure 4). However, for women, increased condom use after HTC had a slightly larger effect in prevention of HIV infections (24 per 1000 in round six) than in reductions in numbers of sexual partners (13 per 1000).

In the client-centric HIV prevention cascades for 2012–13, 15·3%, of sexually active men (223 of 1457) and 36·8% (1000 of 2717) women perceived a risk of HIV infection (p<0·0001; figure 3). 124 (55·6%) of 223 men who felt at risk had taken up HTC compared with 805 (65·2%) of 1234 men who did not (age-adjusted odds ratio 0·66; p=0·005). By contrast, 846 (84·6%) of 1000 women who felt at risk used HTC compared with 1359 (79·1%) of 1717 women who did not feel at risk (1·34; p=0·007). Among those who felt at risk and had taken up HTC, 43·7% of men (49 of 112) and 2·6% of women (21 of 814), had subsequently reduced their number of sexual partners; and 23·2% (26 of 112) and 4·4% (36 of 815) reported an increase in condom use (figure 3).

## Discussion

In this population before 2011, circumcision was not widely available. By 2013, availability had improved somewhat but uptake was still low. In addition to low availability, men at risk did not perceive that risk; even among those who acknowledged a risk, uptake of circumcision was low. Over the past 2 years, circumcision has become more readily available in Zimbabwe and barriers to uptake are being addressed.^19^ However, in the rural populations studied here, acknowledgment and perception of risk of HIV is a substantial challenge, which has many potential causes.^20^

Analysis of HIV prevention cascades can help identify the barriers to effective HIV prevention in populations, which is illustrated by applying different formulations of prevention cascades to data from a population at high risk of HIV acquisition in rural Zimbabwe. This application of the concept for prevention interventions used by susceptible individuals highlights the lack of prevention service delivery, low perceptions of risk, and poor uptake of HIV prevention in rural Zimbabwe. VMMC, condom use, and reduced partner numbers after HTC would be efficacious ways of reducing HIV risk, but they are not widely used in Manicaland.

HIV testing and counselling is often described as a gateway to both HIV treatment and HIV prevention and included in HIV prevention budgets, but without associated changes in behaviour it will not reduce HIV risk. Our analysis shows that, in rural eastern Zimbabwe, HIV testing has little effect as HIV prevention among susceptible people. This is in line with some previous studies of the impact of HIV testing and counselling, which does not change the risk behaviour of HIV-negative people.^21^, ^22^ Over time, the supply and uptake of HTC has improved in both men and women, but the lack of change in partner numbers and condom use associated with HTC undermines its prevention effect. This is particularly notable for women. In such circumstances, inclusion of HTC as an HIV prevention intervention may not be justifiable.

In applying prevention cascades to data, limitations arose from relying on self-reported data on availability including distances to services, uptake of services, and sexual behaviour. Cross-sectional approaches to measuring steps in HIV prevention cascades, as used here, can also be problematic. For example, cross-sectional measurements of perceived risk can be unreliable since perceived risk can lead to VMMC uptake which, in turn, reduces perceived risk, generating higher VMMC in those with no perceived risk. Furthermore the perception of risk, actual risk, duration of risk, and duration of protection provided by an intervention need to be considered when thinking about how to generate a cascade where perception of risk is included. More consideration of such issues will be required when prospectively collecting data to populate prevention cascades.

The data analysed here were not collected with prevention cascade analyses in mind. Other studies could more explicitly include questions relating to each step on the cascades. Prevention cascades are most useful when applied to the underlying population at risk (ie, using population based surveys) rather than those adopting services, since the latter only capture those already taking up services. For planners looking to organise effective HIV prevention this population-level view can come from surveys such as the current HIV Impact Assessments.^23^ However, implementers can still consider in their monitoring and assessment plans the steps of the cascade that they need to improve for their interventions to have effect.

There is no one correct approach to studying HIV prevention cascades, although, for comparability, some agreement on approach would be worthwhile. However, to our knowledge this is the first time that HIV prevention cascades have included data. Other approaches need to be similarly tested with data to assess their utility. In our opinion, it is only by breaking out separate prevention interventions that the prevention cascade can be practically applied, as done here. There are five major limitations to the cascades. First, they only explore reductions in risk of acquiring HIV, not in transmitting HIV, so do not illustrate the importance and role of reduced transmissibility among people with HIV, the impact of which can be assessed with the treatment cascade. Second, they quantify the direct protection provided to susceptible individuals but not the onward protection provided when that individual does not acquire infection; thus they do not quantify the full effect of an intervention but only a minimum level of protection. Third, they do not show the combined protection provided by two or more protection measures. Fourth, they are based on understanding who is at risk of acquiring HIV infection, which can only be inferred from epidemiological data. Generating useful HIV prevention cascades requires a good understanding of patterns of risk of HIV within populations. Fifth, the casades explore the effect of the interventions only for those at risk at the time of the study not for those who could enter the at-risk population in the future and the potential of interventions to change their future behaviours. Potentially, combining cascades in transmission dynamic models could extend our understanding of the impact of our interventions.

The cascades shown here average risk over the adult population and look at the effects of prevention interventions in the time period when the data are collected. Additional insights could be gained by disaggregating the analysis of risk by age (see appendices) or cohort and looking at the age and cohort specific effects of interventions.

In considering the error in measuring cascades, confidence intervals for each step should be based on the binomial distribution as people either do or do not continue to the next step in the cascade. The cascade is a measure of the potential that interventions can have to reduce HIV acquisition and can act as priors in a Bayesian model of the effects of interventions on a population.

The importance of advocacy and monitoring of an HIV prevention cascade has been recognised for some time, but previous attempts have struggled to generate a single prevention cascade covering HIV positive and negative people with multiple prevention approaches. Our concept has been to break down HIV prevention according to both population and prevention approach, so that the important steps can be highlighted and where there are gaps they can be recognised. We can now start to develop an understanding of the more specific problems and what approaches might enable better supply, demand, adherence, or efficacy. This is not to say that delivery of HIV prevention interventions should be siloed as multiple approaches may be necessary and should be combined where this is most efficient. However, it does emphasis the requirements for each approach to have an impact. To illustrate this we have been able to generate HIV prevention cascades for populations in rural Zimbabwe, which illustrate just how far we are from effective HIV prevention targeted at susceptible populations and the scale of the challenges to overcome.

## Figures and Tables

**Table:** Uptake of voluntary medical male circumcision and HIV testing and counselling in HIV-uninfected adults aged 15–54 years in Manicaland, Zimbabwe

			**Men**						**Women**					
			**2009–11**			**2012–13**			**2009–11**			**2012–13**		
			n/N	%	95% CI	n/N	%	95% CI	n/N	%	95% CI	n/N	%	95% CI
**Male circumcision**														
All men														
	Traditional		40/3131	1·28	(0·91–1·74)	63/2245	2·81	(2·16–3·58)	..	..	..	..	..	..
	VMMC		56/3131	1·79	(1·35–2·32)	93/2245	4·14	(3·36–5·05)	..	..	..	..	..	..
Sexually active men														
	VMMC													
		Lifetime	51/1873	2·72	(2·03–3·56)	56/1476	3·79	(2·88–4·90)	..	..	..	..	..	..
		Past 3 years	2/1873	0·11	(0·01–0·39)	24/1476	1·63	(1·04–2·41)	..	..	..	..	..	..
**HIV testing and counselling**														
All respondents														
	Lifetime uptake		867/3154	27·5	(25·9–29·1)	1284/2270	56·6	(54·5–58·6)	2872/4703	61·1	(59·7–62·5)	2625/3297	79·6	(78·2–81·0)
	Past 3 years													
		0	2411/3154	76·4	..	1114/2270	49·1	..	2177/4703	46·3	..	921/3297	27·9	..
		1	505/3154	16·0	..	594/2270	26·2	..	1359/4703	28·9	..	858/3297	26·0	..
		≥2	238/3154	7·6	..	562/2270	24·8	..	1167/4703	24·8	..	1518/3297	46·0	..
Sexually active respondents														
	Lifetime		742/1888	39·3	(37·1–41·6)	1046/1474	71·0	(68·6–73·3)	2742/3793	72·3	(70·8–73·7)	2475/2773	89·3	(88·0–90·4)
	Past 3 years		632/1888	33·5	(31·4–35·7)	941/1474	63·8	(61·3–66·3)	2404/3793	63·4	(61·8–64·9)	2243/2773	80·9	(79·4–82·3)
Behaviour change after HTC*														
Number of sexual partners														
	More		6/563	1·1	..	15/875	1·7	..	6/2198	0·3	..	9/2141	0·4	..
	Same		372/563	6·1	..	594/875	67·9	..	2080/2198	94·6	..	2057/2141	96·1	..
	Fewer		185/563	32·9	..	266/875	30·4	..	112/2198	5·1	..	75/2141	3·5	..
Condom use														
	More		79/563	14·0	..	112/874	12·8	..	90/2199	4·1	..	86/2143	4·0	..
	Same		420/563	74·6	..	637/874	72·9	..	2011/2199	91·5	..	1965/2143	91·7	..
	Fewer		64/563	11·4	..	125/874	14·3	..	98/2199	4·5	..	92/2143	4·3	..

Data given for those who provided responses on the questionnaire, so differ from total sample size owing to missing data.

* HIV testing and counselling in the last 3 years. VMMC=voluntary medical male circumcision. HTC=HIV testing and counselling.
