# Supplementary material for: Providing a conceptual framework for HIV prevention cascades and assessing feasibility of empirical measurement with data from east Zimbabwe: a case study
Source: Lancet HIV. 2016 Jun 27;3(7):e297–306. doi: 10.1016/S2352-3018(16)30039-X (PMC4935672; doi:10.1016/S2352-3018(16)30039-X)
Supplement: Supplementary appendix [file mmc1.pdf]

THE LANCET HIV

Supplementary appendix

This appendix formed part of the original submission and has been peer reviewed. We post it as supplied by the authors.

Supplement to: Garnett GP, Hallett TB, Takaruza A, et al. Providing a conceptual framework for HIV prevention cascades and assessing feasibility of empirical measurement with data from east Zimbabwe: a case study. *Lancet HIV* 2016; **3:** 297–306.

Cascades for HIV prevention: conceptual framework and feasibility of empirical measurement

Geoffrey P Garnett, Timothy B Hallett, Albert Takaruza, James Hargreaves, Rebecca Rhead, Mitchel Warren, Constance Nyamukapa, Simon Gregson

Supplementary Material

HIV incidence estimates

HIV incidence rates were calculated for the eight study sites in Manicaland, eastern Zimbabwe, for which HIV prevention cascades were computed in the main paper and using the same individual eligibility criteria – i.e. adults aged 15-54 years who were resident in and had stayed in a household in the study areas for at least four nights in the month before interview. For the two most recent inter-survey periods (2006-2011 (R4-R5) and 2009-2013 (R5-R6)), HIV incidence was measured in members of the cohort who were uninfected at baseline. For seroconversions between rounds, the date of seroconversion was taken to have occurred midway between the date of baseline interview and the date of follow-up interview. Follow-up time was censored at the date of seroconversion. HIV incidence rates were calculated overall, for men and women who had started sexual activity, and for selected socio-demographic sub-groups. Numbers of seroconversions and person-years of exposure for each period and population group are given in table A1.

HIV incidence in the general population aged 15-54 years in the three-year periods prior to rounds five (R5) and six (R6) of the Manicaland GPCS was 0.80% pa (95% CI, 0.64%-1.00%) and 0.67% (0.53%-0.84%) pa, respectively. Amongst the sexually-experienced population, these rates increase to 0.91% (0.73%-1.13%) and 0.75% (0.60%-0.95%) (table 1A). In both cases – and for each sex – non-statistically-significant reductions in HIV incidence were observed between 2006-2011 and 2009-2013.

Table A1 shows the HIV incidence rates for each period by sex and socio-demographic sub-group. These rates are used to produce the estimates of reductions in HIV incidence achieved through implementation of HIV prevention tools and, for HIV testing, through associated reductions in sexual risk behaviour.

HIV testing and counselling and sexual behaviour change

Figure A1 summarises the data from the last two rounds of the Manicaland GPCS on reported changes in sexual behaviour – reductions in numbers of sexual partners and increases in condom use – following receipt of a negative HIV test result.

In most cases, no change in behaviour was reported and, in many of these cases (especially for women), this is likely to be because the respondent was already abstaining from sexual intercourse or keeping to one regular sexual partner. However, a reasonable minority of men and a small number of women in each round reported a reduction in numbers of sexual partners and/or increased condom use. For example, 30.4% and 12.8% of men in 2012-2013 reported a reduction in number of sexual partners and an increase in condom use, respectively. Some of those who reported a reduction in numbers of sexual partners also reported a *reduction* in condom use; possibly because they were now abstaining or keeping to a single regular partner.

HIV prevention cascades for socio-demographic sub-groups

Comparing HIV prevention cascades between population sub-groups may provide useful information for targeting prevention strategies to those at greatest risk or to those for whom they may be most acceptable.

Tables A2a to A2b show comparisons of HIV prevention cascades for VMMC by age-group, marital status, location (type of study site), and whether or not the respondent had casual sexual partners in the three years before the survey visit. Empirical estimates of actual HIV incidence rates are shown for the same population sub-groups. In 2012-2013, known availability of VMMC services was lowest in widowed men (83.3%), the group that also had the highest rate of HIV incidence (7.2%); however, there were only 28 men in this group (table A2b). Lost opportunities to prevent new HIV infections due to lack of known availability of VMMC services varied from 58.7% in small towns to 71.2% in rural villages and 73.2% in agricultural estates. When services were known to be available, uptake of VMMC was generally low; the highest uptake being seen in divorced and separated men (17.8%, 42/236) – a group with high HIV incidence (2.7%) – and in men aged 45-54 years (16.0%, 43/269; HIV incidence 0.63%).

Tables A2c to A2f show HIV prevention cascades for HTC as an intervention to reduce numbers of sexual partners with comparisons for the same socio-demographic sub-groups. In 2012-2013, for men, lack of knowledge of HTC availability was relatively high in 15-19 year-olds (163/1000) compared to other population sub-groups. Lack of uptake of HTC remained particularly high in divorced men for whom HIV incidence was high (3.0%). Lack of partner reduction was common across the board but greatest amongst married men – possibly because these men were less likely to have casual partners prior to having an HIV test.

For women, lack of knowledge of local availability of HTC had become a minor factor limiting the impact on HTC in preventing HIV infections in those living in the agricultural estates and rural villages but was still a factor for more than 10% of women living in towns – perhaps due to greater population mobility. Lack of uptake of HTC varied by age-group and marital status (highest outside peak childbearing ages and in single and widowed women). Uptake was fairly high in divorced and separated women for whom the greatest numbers of infections were prevented per 1000 at risk (40/1000). Infections prevented per 1000 at risk were also relatively high in single women (32/1000), a group with high HIV incidence (1.6%). Again, only small numbers of HIV-uninfected women reported reducing their number of sexual partners following HTC.

Tables A2g to A2j show HIV prevention cascades for HTC as an intervention to increase condom use again for the same socio-demographic sub-groups. Although this was not the case for men overall, for some high-risk population sub-groups (e.g. divorced and widowed men), HIV infections prevented per 1000 at risk were greater for condom promotion than for partner reduction, possibly due to high levels of casual partners. Also, for women, increased condom use following HTC had a slightly greater impact in preventing HIV (24/1000 in round 6) than reductions in numbers of sexual partners (13/1000). Increased condom use following HTC was slightly higher in divorced and separated women than in other population sub-groups.

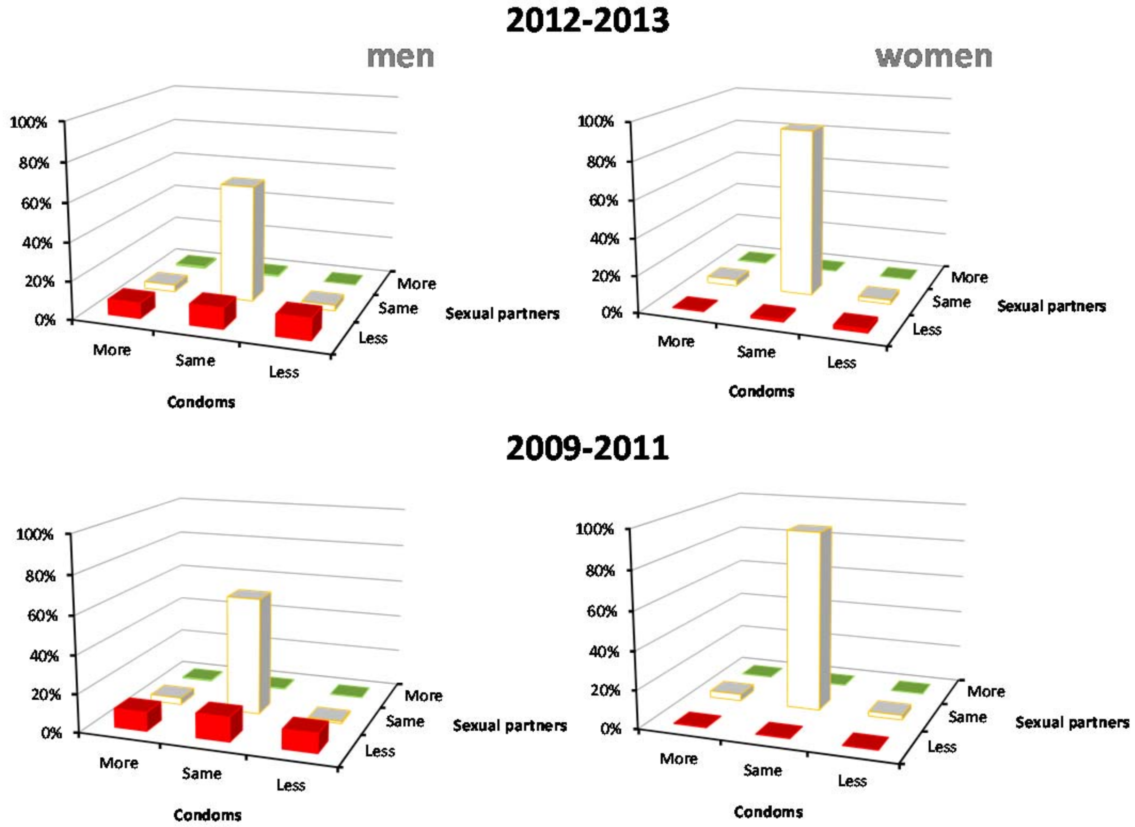

Figure A1: **Changes in numbers of sexual partners and condom use reported by adults aged 15-54 years in Manicaland, Zimbabwe, following receipt of a negative HIV testing and counselling result in the past three years.** (A) Men, 2012-2103. (B) Women, 2012-2013. (C) Men, 2009-011. (D) Women 15-54, 2009-2011.

Characteristic at baseline	2009-2013		2006-2011			
	8 sites		8 sites		12 sites	
	SC/PY	HIV incidence (CI)	SC/PY	HIV incidence (CI)	SC/PY	HIV incidence (CI)
Males, aged 15-54 years						
All	20/2968	0.67 (0.43-1.04)	26/2752	0.94 (0.64-1.39)	28/3854	0.73 (0.50-1.05)
Age-group (years)						
15-19	1/357	0.28 (0.04-1.97)	0/265	0.00	0/388	0.00
20-24	0/420	0.00	3/463	0.64 (0.21-2.01)	3/605	0.50 (0.16-1.54)
25-34	9/1006	0.89 (0.47-1.72)	13/949	1.37 (0.80-2.36)	14/1327	1.05 (0.62-1.78)
35-44	7/708	0.99 (0.47-2.07)	7/575	1.22 (0.58-2.55)	8/824	0.97 (0.49-1.94)
45-54	3/476	0.63 (0.20-1.95)	3/500	0.60 (0.19-1.86)	3/709	0.42 (0.14-1.31)
Marital status						
Single	1/777	0.13 (0.02-0.91)	6/768	0.78 (0.35-1.74)	6/1044	0.57 (0.26-1.28)
Married	15/2086	0.72 (0.43-1.19)	18/1880	0.96 (0.60-1.52)	20/2675	0.75 (0.48-1.16)
Divorced/separated	2/76	2.62 (0.65-10.5)	2/83	2.41 (0.60-9.62)	2/107	1.87 (0.47-0.75)
Widowed	2/28	7.22 (1.80-28.9)	0/21	0.00	0/29	0.00
Location						
Towns	4/832	0.48 (0.18-1.28)	9/607	1.48 (0.77-2.85)	9/607	1.48 (0.77-2.85)
Estates	7/696	1.13 (0.48-2.11)	7/712	0.98 (0.47-2.06)	9/1347	0.67 (0.35-1.28)
Small villages	9/1439	0.63 (0.33-1.20)	10/1433	0.70 (0.38-1.30)	10/1900	0.53 (0.28-0.98)
Migration[b]						
Long-term residents	17/2814	0.60 (0.40-0.97)	21/2367	0.89 (0.58-1.36)	23/3377	0.68 (0.45-1.02)
Recent in-migrants	3/153	1.96 (0.63-6.07)	5/385	1.30 (0.54-3.12)	5/477	1.05 (0.44-2.52)
Females, aged 15-54 years						
All	52/6620	0.79 (0.60-1.03)	51/5735	0.89 (0.68-1.17)	61/8308	0.73 (0.57-0.94)
Age-group (years)						
15-19	1/380	0.26 (0.04-1.87)	3/315	0.95 (0.31-2.95)	3/493	0.61 (0.20-1.89)
20-24	17/908	1.87 (1.16-3.01)	8/868	0.92 (0.46-1.84)	9/1200	0.75 (0.39-1.44)
25-34	16/2070	0.77 (0.47-1.26)	26/1638	1.59 (1.08-2.33)	32/2362	1.36 (0.96-1.92)
35-44	13/1611	0.81 (0.47-1.39)	9/1529	0.59 (0.31-1.13)	11/2167	0.51 (0.28-0.92)
45-54	5/1651	0.30 (0.13-0.73)	5/1384	0.36 (0.15-0.87)	6/2086	0.29 (0.13-0.64)
Marital status						
Single	4/257	1.56 (0.58-4.12)	8/412	1.94 (0.97-3.89)	10/571	1.75 (0.94-3.25)
Married	41/5333	0.77 (0.57-1.04)	30/4278	0.70 (0.49-1.00)	33/6252	0.53 (0.38-0.74)
Divorced/separated	3/376	0.81 (0.26-2.52)	9/403	2.24 (1.16-4.30)	11/572	1.92 (1.06-3.47)
Widowed	4/661	0.60 (0.23-1.61)	4/643	0.62 (0.23-1.66)	7/912	0.77 (0.37-1.61)
Location						
Towns	12/1412	0.85 (0.48-1.50)	18/922	1.95 (1.23-3.10)	18/922	1.95 (1.23-3.10)
Estates	9/1267	0.71 (0.37-1.36)	9/1090	0.83 (0.43-1.59)	15/2130	0.70 (0.42-1.17)
Small villages	31/3940	0.79 (0.55-1.12)	24/3723	0.64 (0.43-0.96)	28/5256	0.53 (0.37-0.77)
Migration[b]						
Long-term residents	43/5987	0.72 (0.53-0.97)	43/5059	0.85 (0.63-1.15)	53/7405	0.72 (0.55-0.94)
Recent in-migrants	9/632	1.42 (0.74-2.73)	8/675	1.18 (0.59-2.37)	8/903	0.89 (0.44-1.77)
Males and females combined						
All	72/9587	0.75 (0.60-0.95)	77/8487	0.91 (0.73-1.13)	89/12163	0.73 (0.59-0.90)

[a] Amongst regular household members at baseline who had stayed in the household for at least four nights in the last month before interview
[b] Migration into the area within the previous three years (i.e. prior to baseline)
New infections assumed to take place half-way between the dates of interview at the last round tested negative and the first round tested positive
CI, 95% confidence interval; PY, person years; SC, seroconverters

Table A1: **HIV incidence amongst sexually-experienced adult (15-54 yrs) residents, Manicaland, Zimbabwe, 2006-2013[a]**

Population sub-group		Infections Potential	Infections not prevented due to lack of: Availability	Uptake	Adherence	Efficacy	Infections Not prevented	Prevented	HIV incidence Potential	Actual	Prevented	PY
All men	Cumulative	1000	971	982	982	989	989	11	0.74%	0.73%	0.01%	3547
	This stage of cascade	1000	971	11	0	7						
Sexually-active men	Cumulative	1000	964	973	973	984	984	16	0.96%	0.94%	0.02%	2752
	This stage of cascade	1000	964	9	0	11						
Age-group												
15-19	Cumulative	1000	967	983	983	990	990	10	0.00%	0.00%	0.00%	265
	This stage of cascade	1000	967	16	0	7						
20-24	Cumulative	1000	972	979	979	987	987	13	0.65%	0.64%	0.01%	463
	This stage of cascade	1000	972	7	0	8						
25-34	Cumulative	1000	969	975	975	985	985	15	1.39%	1.37%	0.02%	949
	This stage of cascade	1000	969	6	0	10						
35-44	Cumulative	1000	958	975	975	985	985	15	1.24%	1.22%	0.02%	575
	This stage of cascade	1000	958	17	0	10						
45-54	Cumulative	1000	950	954	954	973	973	27	0.62%	0.60%	0.02%	500
	This stage of cascade	1000	950	4	0	19						
Marital status												
Single	Cumulative	1000	972	979	979	987	987	13	0.79%	0.78%	0.01%	768
	This stage of cascade	1000	972	7	0	8						
Married	Cumulative	1000	969	975	975	985	985	15	0.98%	0.96%	0.02%	1880
	This stage of cascade	1000	969	6	0	10						
Divorced or separated	Cumulative	1000	958	975	975	985	985	15	2.52%	2.41%	0.11%	83
	This stage of cascade	1000	958	17	0	10						
Widowed	Cumulative	1000	950	954	954	973	973	27	0.00%	0.00%	0.00%	21
	This stage of cascade	1000	950	4	0	19						
Location												
Towns	Cumulative	1000	945	972	972	983	983	17	1.51%	1.48%	0.03%	607
	This stage of cascade	1000	945	27	0	11						
Estates	Cumulative	1000	962	962	962	977	977	23	1.00%	0.98%	0.02%	712
	This stage of cascade	1000	962	0	0	15						
Small villages	Cumulative	1000	974	978	978	987	987	13	0.71%	0.70%	0.01%	1433
	This stage of cascade	1000	974	4	0	9						
Casual sexual partners												
≥1 in last 3 years	Cumulative	1000	964	973	973	984	984	16	1.47%	1.45%	0.02%	689
	This stage of cascade	1000	964	9	0	11						
None in last 3 years	Cumulative	1000	963	972	972	983	983	17	0.79%	0.78%	0.01%	2060
	This stage of cascade	1000	963	9	0	11						

Table A2a: **HIV prevention cascades for VMMC by population sub-group, Manicaland, Zimbabwe, 2009-2011**

Population sub-group		Infections Potential	Infections not prevented due to lack of: Availability	Uptake	Adherence	Efficacy	Infections Not prevented	Prevented	HIV incidence Potential	Actual	Prevented	PY
All men	Cumulative	1000	682	959	959	976	976	24	0.55%	0.53%	0.01%	3762
	This stage of cascade	1000	682	277	0	17						
Sexually-active men	Cumulative	1000	684	962	962	977	977	23	0.69%	0.67%	0.02%	2968
	This stage of cascade	1000	684	278	0	15						
Age-group												
15-19	Cumulative	1000	697	955	955	973	973	27	0.29%	0.28%	0.01%	357
	This stage of cascade	1000	697	258	0	18						
20-24	Cumulative	1000	671	988	988	993	993	7	0.00%	0.00%	0.00%	420
	This stage of cascade	1000	671	317	0	5						
25-34	Cumulative	1000	679	961	961	977	977	23	0.91%	0.89%	0.02%	1006
	This stage of cascade	1000	679	282	0	16						
35-44	Cumulative	1000	672	951	951	970	970	30	1.02%	0.99%	0.03%	708
	This stage of cascade	1000	672	279	0	19						
45-54	Cumulative	1000	731	957	957	974	974	26	0.65%	0.63%	0.02%	476
	This stage of cascade	1000	731	226	0	17						
Marital status												
Single	Cumulative	1000	645	967	967	980	980	20	0.13%	0.13%	0.00%	777
	This stage of cascade	1000	645	322	0	13						
Married	Cumulative	1000	689	960	960	976	976	24	0.74%	0.72%	0.02%	2086
	This stage of cascade	1000	689	271	0	16						
Divorced or separated	Cumulative	1000	764	958	958	975	975	25	2.69%	2.62%	0.07%	76
	This stage of cascade	1000	764	194	0	17						
Widowed	Cumulative	1000	833	1000	1000	1000	1000	0	7.22%	7.22%	0.00%	28
	This stage of cascade	1000	833	167	0	0						
Location												
Towns	Cumulative	1000	587	955	955	973	973	27	0.49%	0.48%	0.01%	832
	This stage of cascade	1000	587	368	0	18						
Estates	Cumulative	1000	732	971	971	983	983	17	1.15%	1.13%	0.02%	696
	This stage of cascade	1000	732	239	0	12						
Small villages	Cumulative	1000	712	962	962	977	977	23	0.65%	0.63%	0.02%	1439
	This stage of cascade	1000	712	250	0	15						

Table A2b: **HIV prevention cascades for VMMC by population sub-group, Manicaland, Zimbabwe, 2012-2013**

Population sub-group		Infections potential	Infections not prevented due to lack of: HTC availability	HTC uptake	Partner reduction	Efficacy	Infections Not prevented	Prevented	HIV incidence Potential	Actual	Prevented	PY
All men	Cumulative	1000	361	793	938	969	969	31	0.75%	0.73%	0.02%	3547
	This stage of cascade	1000	361	432	145	31						
Sexually-active men	Cumulative	1000	270	702	902	951	951	49	0.99%	0.94%	0.05%	2752
	This stage of cascade	1000	270	432	200	49						
Age-group												
15-19	Cumulative	1000	328	853	968	984	984	16	0.00%	0.00%	0.00%	265
	This stage of cascade	1000	328	525	115	16						
20-24	Cumulative	1000	306	705	910	955	955	45	0.67%	0.64%	0.03%	463
	This stage of cascade	1000	306	399	205	45						
25-34	Cumulative	1000	234	673	889	945	945	55	1.45%	1.37%	0.08%	949
	This stage of cascade	1000	234	439	216	56						
35-44	Cumulative	1000	260	702	903	951	951	49	1.28%	1.22%	0.06%	575
	This stage of cascade	1000	260	442	201	48						
45-54	Cumulative	1000	337	746	914	957	957	43	0.63%	0.60%	0.03%	500
	This stage of cascade	1000	337	409	168	43						
Marital status												
Single	Cumulative	1000	311	740	915	958	958	42	0.82%	0.78%	0.04%	768
	This stage of cascade	1000	311	429	175	43						
Married	Cumulative	1000	260	693	901	950	950	50	1.01%	0.96%	0.05%	1880
	This stage of cascade	1000	260	433	208	49						
Divorced or separated	Cumulative	1000	319	750	889	945	945	55	2.55%	2.41%	0.14%	83
	This stage of cascade	1000	319	431	139	56						
Widowed	Cumulative	1000	136	591	773	887	887	113	0.00%	0.00%	0.00%	21
	This stage of cascade	1000	136	455	182	114						
Location												
Towns	Cumulative	1000	235	678	870	935	935	65	1.58%	1.48%	0.10%	607
	This stage of cascade	1000	235	443	192	65						
Estates	Cumulative	1000	406	788	919	959	959	41	1.02%	0.98%	0.04%	712
	This stage of cascade	1000	406	382	131	40						
Small villages	Cumulative	1000	221	672	910	955	955	45	0.73%	0.70%	0.03%	1433
	This stage of cascade	1000	221	451	238	45						
Casual sexual partners												
≥1 in last 3 years	Cumulative	1000	270	693	838	919	919	81	1.58%	1.45%	0.13%	689
	This stage of cascade	1000	270	423	145	81						
None in last 3 years	Cumulative	1000	269	704	926	963	963	37	0.81%	0.78%	0.03%	2060
	This stage of cascade	1000	269	435	222	37						

Table A2c: **HIV prevention cascades for HTC and sexual partner reduction for men by population sub-group, Manicaland, Zimbabwe, 2009-2011**

Population sub-group		Infections potential	Infections not prevented due to lack of: HTC availability	HTC uptake	Partner reduction	Efficacy	Infections Not prevented	Prevented	HIV incidence Potential	Actual	Prevented	PY
All women	Cumulative	1000	204	509	975	987	987	13	0.85%	0.84%	0.01%	6076
	This stage of cascade	1000	204	305	466	12						
Sexually-active women	Cumulative	1000	151	420	970	985	985	15	0.90%	0.89%	0.01%	5735
	This stage of cascade	1000	151	269	550	15						
Age-group												
15-19	Cumulative	1000	239	428	967	983	983	17	0.97%	0.95%	0.02%	315
	This stage of cascade	1000	239	189	539	16						
20-24	Cumulative	1000	159	329	980	990	990	10	0.93%	0.92%	0.01%	868
	This stage of cascade	1000	159	170	651	10						
25-34	Cumulative	1000	104	335	964	982	982	18	1.62%	1.59%	0.03%	1638
	This stage of cascade	1000	104	231	629	18						
35-44	Cumulative	1000	145	466	965	982	982	18	0.60%	0.59%	0.01%	1529
	This stage of cascade	1000	145	321	499	17						
45-54	Cumulative	1000	194	584	979	990	990	10	0.36%	0.36%	0.00%	1384
	This stage of cascade	1000	194	390	395	11						
Marital status												
Single	Cumulative	1000	281	588	965	983	983	17	1.98%	1.94%	0.04%	412
	This stage of cascade	1000	281	307	377	18						
Married	Cumulative	1000	143	393	973	987	987	13	0.71%	0.70%	0.01%	4278
	This stage of cascade	1000	143	250	580	14						
Divorced or separated	Cumulative	1000	153	482	943	972	972	28	2.31%	2.24%	0.07%	403
	This stage of cascade	1000	153	329	461	29						
Widowed	Cumulative	1000	176	552	977	989	989	11	0.63%	0.62%	0.01%	643
	This stage of cascade	1000	176	376	425	12						
Location												
Towns	Cumulative	1000	102	355	979	990	990	10	1.97%	1.95%	0.02%	922
	This stage of cascade	1000	102	253	624	11						
Estates	Cumulative	1000	233	495	977	989	989	11	0.84%	0.83%	0.01%	1090
	This stage of cascade	1000	233	262	482	12						
Small villages	Cumulative	1000	142	420	965	982	982	18	0.65%	0.64%	0.01%	3723
	This stage of cascade	1000	142	278	545	17						
Casual sexual partners												
≥1 in last 3 years	Cumulative	1000	214	498	949	975	975	25	4.36%	4.25%	0.11%	259
	This stage of cascade	1000	214	284	451	26						
None in last 3 years	Cumulative	1000	147	416	972	986	986	14	0.74%	0.73%	0.01%	5464
	This stage of cascade	1000	147	269	556	14						

Table A2d: **HIV prevention cascades for HTC and sexual partner reduction for women by population sub-group, Manicaland, Zimbabwe, 2009-2011**

Population sub-group		Infections potential	Infections not prevented due to lack of: HTC availability	HTC uptake	Partner reduction	Efficacy	Infections Not prevented	Prevented	HIV incidence Potential	Actual	Prevented	PY
All men	Cumulative	1000	152	525	871	935	935	65	0.57%	0.53%	0.04%	3762
	This stage of cascade	1000	152	373	346	64						
Sexually-active men	Cumulative	1000	84	406	820	910	910	90	0.74%	0.67%	0.07%	2968
	This stage of cascade	1000	84	322	414	90						
Age-group												
15-19	Cumulative	1000	163	558	860	930	930	70	0.30%	0.28%	0.02%	357
	This stage of cascade	1000	163	395	302	70						
20-24	Cumulative	1000	97	436	834	917	917	83	0.00%	0.00%	0.00%	420
	This stage of cascade	1000	97	339	398	83						
25-34	Cumulative	1000	84	371	790	895	895	105	0.99%	0.89%	0.10%	1006
	This stage of cascade	1000	84	287	419	105						
35-44	Cumulative	1000	48	405	835	918	918	82	1.08%	0.99%	0.09%	708
	This stage of cascade	1000	48	357	430	83						
45-54	Cumulative	1000	98	406	841	920	920	80	0.68%	0.63%	0.05%	476
	This stage of cascade	1000	98	308	435	79						
Marital status												
Single	Cumulative	1000	104	530	828	914	914	86	0.14%	0.13%	0.01%	777
	This stage of cascade	1000	104	426	298	86						
Married	Cumulative	1000	77	360	823	912	912	88	0.79%	0.72%	0.07%	2086
	This stage of cascade	1000	77	283	463	89						
Divorced or separated	Cumulative	1000	96	521	754	877	877	123	2.99%	2.62%	0.37%	76
	This stage of cascade	1000	96	425	233	123						
Widowed	Cumulative	1000	0	250	667	834	834	166	8.66%	7.22%	1.44%	28
	This stage of cascade	1000	0	250	417	167						
Location												
Towns	Cumulative	1000	87	412	810	905	905	95	0.53%	0.48%	0.05%	832
	This stage of cascade	1000	87	325	398	95						
Estates	Cumulative	1000	108	418	816	908	908	92	1.25%	1.13%	0.12%	696
	This stage of cascade	1000	108	310	398	92						
Small villages	Cumulative	1000	72	397	826	913	913	87	0.69%	0.63%	0.06%	1439
	This stage of cascade	1000	72	325	429	87						
Casual sexual partners												
≥1 in last 3 years	Cumulative	1000	89	451	770	885	885	115	1.26%	1.11%	0.14%	988
	This stage of cascade	1000	89	362	319	115						
None in last 3 years	Cumulative	1000	80	377	847	924	924	76	0.45%	0.41%	0.03%	1696
	This stage of cascade	1000	80	297	470	77						

Table A2e: **HIV prevention cascades for HTC and sexual partner reduction for men by population sub-group, Manicaland, Zimbabwe, 2012-2013**

Population sub-group		Infections	Infections not prevented due to lack of:				Infections		HIV incidence			
		potential	HTC availability	HTC uptake	Partner reduction	Efficacy	Not prevented	Prevented	Potential	Actual	Prevented	PY
All women	Cumulative	1000	88	314	976	988	988	12	0.75%	0.74%	0.01%	7047
	This stage of cascade	1000	88	226	662	12						
Sexually-active women	Cumulative	1000	53	227	973	987	987	13	0.80%	0.79%	0.01%	6620
	This stage of cascade	1000	53	174	746	14						
Age-group												
15-19	Cumulative	1000	94	214	965	982	982	18	0.27%	0.26%	0.01%	380
	This stage of cascade	1000	94	120	751	17						
20-24	Cumulative	1000	40	106	978	989	989	11	1.89%	1.87%	0.02%	908
	This stage of cascade	1000	40	66	872	11						
25-34	Cumulative	1000	41	171	973	986	986	14	0.78%	0.77%	0.01%	2070
	This stage of cascade	1000	41	130	802	13						
35-44	Cumulative	1000	49	250	974	987	987	13	0.82%	0.81%	0.01%	1611
	This stage of cascade	1000	49	201	724	13						
45-54	Cumulative	1000	69	401	969	984	984	16	0.31%	0.30%	0.01%	1651
	This stage of cascade	1000	69	332	568	15						
Marital status												
Single	Cumulative	1000	90	385	936	968	968	32	1.61%	1.56%	0.05%	257
	This stage of cascade	1000	90	295	551	32						
Married	Cumulative	1000	45	202	980	990	990	10	0.78%	0.77%	0.01%	5333
	This stage of cascade	1000	45	157	778	10						
Divorced or separated	Cumulative	1000	88	246	920	960	960	40	0.84%	0.81%	0.03%	376
	This stage of cascade	1000	88	158	674	40						
Widowed	Cumulative	1000	79	401	962	981	981	19	0.61%	0.60%	0.01%	661
	This stage of cascade	1000	79	322	561	19						
Location												
Towns	Cumulative	1000	113	279	989	994	994	6	0.86%	0.85%	0.01%	1412
	This stage of cascade	1000	113	166	710	5						
Estates	Cumulative	1000	53	204	954	977	977	23	0.73%	0.71%	0.02%	1267
	This stage of cascade	1000	53	151	750	23						
Small villages	Cumulative	1000	27	212	973	987	987	13	0.80%	0.79%	0.01%	3940
	This stage of cascade	1000	27	185	761	14						
Casual sexual partners												
≥1 in last 3 years	Cumulative	1000	53	188	932	966	966	34	2.65%	2.56%	0.09%	234
	This stage of cascade	1000	53	135	744	34						
None in last 3 years	Cumulative	1000	53	223	974	987	987	13	0.71%	0.70%	0.01%	5874
	This stage of cascade	1000	53	170	751	13						

Table A2f: **HIV prevention cascades for HTC and sexual partner reduction for women by population sub-group, Manicaland, Zimbabwe, 2012-2013**

Population sub-group		Infections potential	Infections not prevented due to lack of: HTC availability	HTC uptake	Increased condom use	Efficacy	Infections Not prevented	Prevented	HIV incidence Potential	Actual	Prevented	PY
All men	Cumulative	1000	361	793	975	980	980	20	0.75%	0.73%	0.01%	3547
	This stage of cascade	1000	361	432	182	5						
Sexually-active men	Cumulative	1000	270	702	958	966	966	34	0.97%	0.94%	0.03%	2752
	This stage of cascade	1000	270	432	256	8						
Age-group												
15-19	Cumulative	1000	328	853	984	987	987	13	0.00%	0.00%	0.00%	265
	This stage of cascade	1000	328	525	131	3						
20-24	Cumulative	1000	306	705	945	956	956	44	0.67%	0.64%	0.03%	463
	This stage of cascade	1000	306	399	240	11						
25-34	Cumulative	1000	234	673	955	964	964	36	1.42%	1.37%	0.05%	949
	This stage of cascade	1000	234	439	282	9						
35-44	Cumulative	1000	260	702	964	971	971	29	1.26%	1.22%	0.04%	575
	This stage of cascade	1000	260	442	262	7						
45-54	Cumulative	1000	337	746	965	972	972	28	0.62%	0.60%	0.02%	500
	This stage of cascade	1000	337	409	219	7						
Marital status												
Single	Cumulative	1000	311	740	915	932	932	68	0.79%	0.78%	0.01%	768
	This stage of cascade	1000	311	429	175	17						
Married	Cumulative	1000	260	693	970	976	976	24	1.01%	0.96%	0.05%	1880
	This stage of cascade	1000	260	433	277	6						
Divorced or separated	Cumulative	1000	319	750	931	945	945	55	2.55%	2.41%	0.14%	83
	This stage of cascade	1000	319	431	181	14						
Widowed	Cumulative	1000	136	591	864	891	891	109	0.00%	0.00%	0.00%	21
	This stage of cascade	1000	136	455	273	27						
Location												
Towns	Cumulative	1000	235	678	947	958	958	42	1.55%	1.48%	0.07%	607
	This stage of cascade	1000	235	443	269	11						
Estates	Cumulative	1000	406	788	971	977	977	23	1.00%	0.98%	0.02%	712
	This stage of cascade	1000	406	382	183	6						
Small villages	Cumulative	1000	221	672	957	966	966	34	0.73%	0.70%	0.03%	1433
	This stage of cascade	1000	221	451	285	9						
Casual sexual partners												
≥1 in last 3 years	Cumulative	1000	270	693	912	930	930	70	1.56%	1.45%	0.11%	689
	This stage of cascade	1000	270	423	219	18						
None in last 3 years	Cumulative	1000	269	704	976	981	981	19	0.80%	0.78%	0.02%	2060
	This stage of cascade	1000	269	435	272	5						

Table A2g: **HIV prevention cascades for HTC and increased condom use for men by population sub-group, Manicaland, Zimbabwe, 2009-2011**

Population sub-group		Infections potential	Infections not prevented due to lack of: HTC availability	HTC uptake	Increased condom use	Efficacy	Infections Not prevented	Prevented	HIV incidence Potential	Actual	Prevented	PY
All women	Cumulative	1000	204	509	980	984	984	16	0.85%	0.84%	0.01%	6076
	This stage of cascade	1000	204	305	471	4						
Sexually-active women	Cumulative	1000	151	420	976	981	981	19	0.91%	0.89%	0.02%	5735
	This stage of cascade	1000	151	269	556	5						
Age-group												
15-19	Cumulative	1000	239	428	984	987	987	13	0.96%	0.95%	0.01%	315
	This stage of cascade	1000	239	189	556	3						
20-24	Cumulative	1000	159	329	987	990	990	10	0.93%	0.92%	0.01%	868
	This stage of cascade	1000	159	170	658	3						
25-34	Cumulative	1000	104	335	977	982	982	18	1.62%	1.59%	0.03%	1638
	This stage of cascade	1000	104	231	642	5						
35-44	Cumulative	1000	145	466	968	974	974	26	0.61%	0.59%	0.02%	1529
	This stage of cascade	1000	145	321	502	6						
45-54	Cumulative	1000	194	584	972	978	978	22	0.37%	0.36%	0.01%	1384
	This stage of cascade	1000	194	390	388	6						
Marital status												
Single	Cumulative	1000	281	588	983	987	987	13	1.97%	1.94%	0.03%	412
	This stage of cascade	1000	281	307	395	4						
Married	Cumulative	1000	143	393	974	979	979	21	0.72%	0.70%	0.02%	4278
	This stage of cascade	1000	143	250	581	5						
Divorced or separated	Cumulative	1000	153	482	974	979	979	21	2.29%	2.24%	0.05%	403
	This stage of cascade	1000	153	329	492	5						
Widowed	Cumulative	1000	176	552	1000	1000	1000	0	0.62%	0.62%	0.00%	643
	This stage of cascade	1000	176	376	448	0						
Location												
Towns	Cumulative	1000	102	355	975	980	980	20	1.99%	1.95%	0.04%	922
	This stage of cascade	1000	102	253	620	5						
Estates	Cumulative	1000	233	495	964	971	971	29	0.86%	0.83%	0.03%	1090
	This stage of cascade	1000	233	262	469	7						
Small villages	Cumulative	1000	142	420	980	984	984	16	0.65%	0.64%	0.01%	3723
	This stage of cascade	1000	142	278	560	4						
Casual sexual partners												
≥1 in last 3 years	Cumulative	1000	214	498	963	970	970	30	4.38%	4.25%	0.13%	259
	This stage of cascade	1000	214	284	465	7						
None in last 3 years	Cumulative	1000	147	416	977	982	982	18	0.74%	0.73%	0.01%	5464
	This stage of cascade	1000	147	269	561	5						

Table A2h: **HIV prevention cascades for HTC and increased condom use for women by population sub-group, Manicaland, Zimbabwe, 2009-2011**

Population sub-group		Infections potential	Infections not prevented due to lack of: HTC availability	HTC uptake	Increased condom use	Efficacy	Infections Not prevented	Prevented	HIV incidence Potential	Actual	Prevented	PY
All men	Cumulative	1000	152	525	951	961	961	39	0.55%	0.53%	0.02%	3762
	This stage of cascade	1000	152	373	426	10						
Sexually-active men	Cumulative	1000	84	406	924	939	939	61	0.71%	0.67%	0.04%	2968
	This stage of cascade	1000	84	322	518	15						
Age-group												
15-19	Cumulative	1000	163	558	872	898	898	102	0.31%	0.28%	0.03%	357
	This stage of cascade	1000	163	395	314	26						
20-24	Cumulative	1000	97	436	894	915	915	85	0.00%	0.00%	0.00%	420
	This stage of cascade	1000	97	339	458	21						
25-34	Cumulative	1000	84	371	936	949	949	51	0.94%	0.89%	0.05%	1006
	This stage of cascade	1000	84	287	565	13						
35-44	Cumulative	1000	48	405	939	951	951	49	1.04%	0.99%	0.05%	708
	This stage of cascade	1000	48	357	534	12						
45-54	Cumulative	1000	98	406	920	936	936	64	0.67%	0.63%	0.04%	476
	This stage of cascade	1000	98	308	514	16						
Marital status												
Single	Cumulative	1000	104	530	864	891	891	109	0.15%	0.13%	0.02%	777
	This stage of cascade	1000	104	426	334	27						
Married	Cumulative	1000	77	360	951	961	961	39	0.75%	0.72%	0.03%	2086
	This stage of cascade	1000	77	283	591	10						
Divorced or separated	Cumulative	1000	96	521	822	858	858	142	3.06%	2.62%	0.44%	76
	This stage of cascade	1000	96	425	301	36						
Widowed	Cumulative	1000	0	250	750	800	800	200	9.03%	7.22%	1.81%	28
	This stage of cascade	1000	0	250	500	50						
Location												
Towns	Cumulative	1000	87	412	940	952	952	48	0.50%	0.48%	0.02%	832
	This stage of cascade	1000	87	325	528	12						
Estates	Cumulative	1000	108	418	900	920	920	80	1.23%	1.13%	0.10%	696
	This stage of cascade	1000	108	310	482	20						
Small villages	Cumulative	1000	72	397	927	942	942	58	0.67%	0.63%	0.04%	1439
	This stage of cascade	1000	72	325	530	15						
Casual sexual partners												
≥1 in last 3 years	Cumulative	1000	89	451	868	894	894	106	1.24%	1.11%	0.13%	988
	This stage of cascade	1000	89	362	417	26						
None in last 3 years	Cumulative	1000	80	377	957	966	966	34	0.43%	0.41%	0.02%	1696
	This stage of cascade	1000	80	297	580	9						

Table A2i: **HIV prevention cascades for HTC and increased condom use for men by population sub-group, Manicaland, Zimbabwe, 2012-2013**

Population sub-group		Infections potential	Infections not prevented due to lack of: HTC availability	HTC uptake	Increased condom use	Efficacy	Infections Not prevented	Prevented	HIV incidence Potential	Actual	Prevented	PY
All women	Cumulative	1000	88	314	974	979	979	21	0.76%	0.74%	0.02%	7047
	This stage of cascade	1000	88	226	660	5						
Sexually-active women	Cumulative	1000	53	227	970	976	976	24	0.81%	0.79%	0.02%	6620
	This stage of cascade	1000	53	174	743	6						
Age-group												
15-19	Cumulative	1000	94	214	995	996	996	4	0.26%	0.26%	0.00%	380
	This stage of cascade	1000	94	120	781	1						
20-24	Cumulative	1000	40	106	985	988	988	12	1.89%	1.87%	0.02%	908
	This stage of cascade	1000	40	66	879	3						
25-34	Cumulative	1000	41	171	973	978	978	22	0.79%	0.77%	0.02%	2070
	This stage of cascade	1000	41	130	802	5						
35-44	Cumulative	1000	49	250	951	961	961	39	0.84%	0.81%	0.03%	1611
	This stage of cascade	1000	49	201	701	10						
45-54	Cumulative	1000	69	401	956	965	965	35	0.31%	0.30%	0.01%	1651
	This stage of cascade	1000	69	332	555	9						
Marital status												
Single	Cumulative	1000	90	385	962	970	970	30	1.61%	1.56%	0.05%	257
	This stage of cascade	1000	90	295	577	8						
Married	Cumulative	1000	45	202	974	979	979	21	0.79%	0.77%	0.02%	5333
	This stage of cascade	1000	45	157	772	5						
Divorced or separated	Cumulative	1000	88	246	916	933	933	67	0.87%	0.81%	0.06%	376
	This stage of cascade	1000	88	158	670	17						
Widowed	Cumulative	1000	79	401	966	973	973	27	0.62%	0.60%	0.02%	661
	This stage of cascade	1000	79	322	565	7						
Location												
Towns	Cumulative	1000	113	279	979	983	983	17	0.86%	0.85%	0.01%	1412
	This stage of cascade	1000	113	166	700	4						
Estates	Cumulative	1000	53	204	956	965	965	35	0.74%	0.71%	0.03%	1267
	This stage of cascade	1000	53	151	752	9						
Small villages	Cumulative	1000	27	212	969	975	975	25	0.81%	0.79%	0.02%	3940
	This stage of cascade	1000	27	185	757	6						
Casual sexual partners												
≥1 in last 3 years	Cumulative	1000	53	188	895	916	916	84	2.80%	2.56%	0.24%	234
	This stage of cascade	1000	53	135	707	21						
None in last 3 years	Cumulative	1000	53	223	972	978	978	22	0.71%	0.70%	0.02%	5874
	This stage of cascade	1000	53	170	749	6						

Table A2j: **HIV prevention cascades for HTC and increased condom use for women by population sub-group, Manicaland, Zimbabwe, 2012-2013**
